# Exposure to the parents’ speech is positively associated with preterm infant’s face preference

**DOI:** 10.1038/s41390-024-03239-8

**Published:** 2024-05-23

**Authors:** Anette Aija, Jukka Leppänen, Laura Aarnos, Mirka Hyvönen, Eva Ståhlberg-Forsén, Sari Ahlqvist-Björkroth, Suvi Stolt, Liis Toome, Liisa Lehtonen

**Affiliations:** 1 University of Turku, Turku, Finland; 2 Department of Neonatal and Infant Medicine, Tallinn Children’s Hospital, Tallinn, Estonia; 3 Department of Psychology and Speech-Language Pathology, University of Turku, Turku, Finland; 4 Department of Psychology and Logopedics, University of Helsinki, Helsinki, Finland; 5 Department of Pediatrics, Turku University Hospital, Turku, Finland

## Abstract

**Background:**

The parents’ presence and involvement in neonatal care is a promising approach to improve preterm infants’ neurodevelopmental outcomes. We examined whether exposure to the parents’ speech is associated with the preterm infant’s social-cognitive development.

**Methods:**

The study included infants born before 32 gestational weeks in two neonatal units. Each infant’s language environment was assessed from 16-hour recordings using Language Environment Analysis (LENA®). Parental presence was assessed with Closeness Diary for 14 days during the hospital stay. Attention to faces and non-face patterns was measured at the corrected age of seven months using an eye-tracking disengagement test.

**Results:**

A total of 63 preterm infants were included. Infants were less likely to disengage their attention from faces (*M* = 0.55, SD = 0.26) than non-face patterns (*M* = 0.24, SD = 0.22), *p* < 0.001, *d* = 0.84. Exposure to the parents’ speech during the neonatal period was positively correlated with the preference for faces over non-face patterns (*r*_s_ = 0.34, *p* = 0.009) and with the preference for parents over unfamiliar faces (*r*_s_ = 0.28, *p* = 0.034).

**Conclusion:**

The exposure to the parents’ speech during neonatal hospital care is a potential early marker for later social development in preterm infants.

**Impact:**

- The exposure to the parents’ speech during neonatal intensive care is a potential early marker for optimal social-cognitive development in preterm infants.
- This is the first study to show an association between parental vocal contact during neonatal intensive care and early social development (i.e., face preference), measured at seven months of corrected age.
- Our findings suggest that we should pay attention to the parents’ vocal contact with their child in the neonatal intensive care unit and identify need for tailored support for face-to-face and vocal contact.

## Introduction

Very preterm infants have an increased risk for developmental problems, including autism spectrum disorders,^1–3^ delays in language development,^4^ visual attention dysfunction^5,6^ and socio-emotional problems.^7^ The parents’ presence and involvement in infant care during hospital treatment is a promising approach to improve later outcomes.^8–12^ Some reviews conclude that exposure to human voices during neonatal intensive care supports the infant’s physiological and behavioral stability.^13,14^ When recorded maternal voice was administered to preterm infants by bone conduction, the visual and auditory orientation, and neurobehavioral development improved at three months of age.^15^ Although a positive association to cognitive and language development at 18 months of corrected age has been found,^16^ another study showed a negative association between the amount of adult talk in the neonatal intensive care unit (NICU) and very preterm children’s language processing ability at the same age.^17^

Compared to term infants, preterm infants demonstrate reduced attention to faces and reduced response to social stimuli,^18^ deficits in attention control^19^, and impaired social attention.^20,21^ Moreover, a delay in visual attention at one year of corrected age is associated with lower cognitive scores at two years of corrected age in very preterm infants.^22^ Infants, even as young as three months of age, prefer to look at faces more than non-face patterns or objects,^23–25^ and this attentional bias for faces can be objectively studied by using eye-tracking technologies. Relatively stronger bias to attend to faces or eyes in infancy has been associated with higher levels of prosocial behavior^26^ as well as a lower risk for autism^27^ later in childhood. Reduced visual attention has also been suggested to predict autistic features^27–29^ and a delay in language acquisition.^30,31^

Many preterm infants experience too little exposure to human speech and too much exposure to noise during intensive care.^32,33^ Some studies^15,16,34^ have demonstrated beneficial effects of language exposure on preterm infants’ development. However, the data on short- and long-term effects are still controversial and inconclusive due to inconsistencies and limited sample sizes in studies on this topic.^17,32,35^ There is also a paucity of studies in units that have implemented modern family-centered care and where parents can spend long times daily with their infants. Therefore, we chose to study the exposure to the parents’ speech (i.e., number of words) in very preterm infants cared for in family-centered NICUs, which are expected to have high levels of parental presence. This setting allowed us to study whether parental vocal contact associated with social-cognitive development in preterm infants. We hypothesized that exposure to the parents’ speech during neonatal care is positively associated with the preference for faces over non-face patterns at seven months of corrected age. We also examined whether exposure to the parents’ speech associated with a stronger preference for parent versus unfamiliar adult faces.

## Methods

### Subjects

Participants in this study are part of the APPLE Study (Auditory environment by Parents of Preterm infant; Language development and Eye-movements, Clinical Trials ID NCT04826978), a prospective longitudinal study conducted in a level IIIB NICU (as defined by the American Academy of Pediatrics^36^) in Turku, Finland, and a level II NICU in Tallinn, Estonia. The participants were recruited between 2017 and 2020. The follow-up continued until the corrected age of two years.

**Fig. 1:**
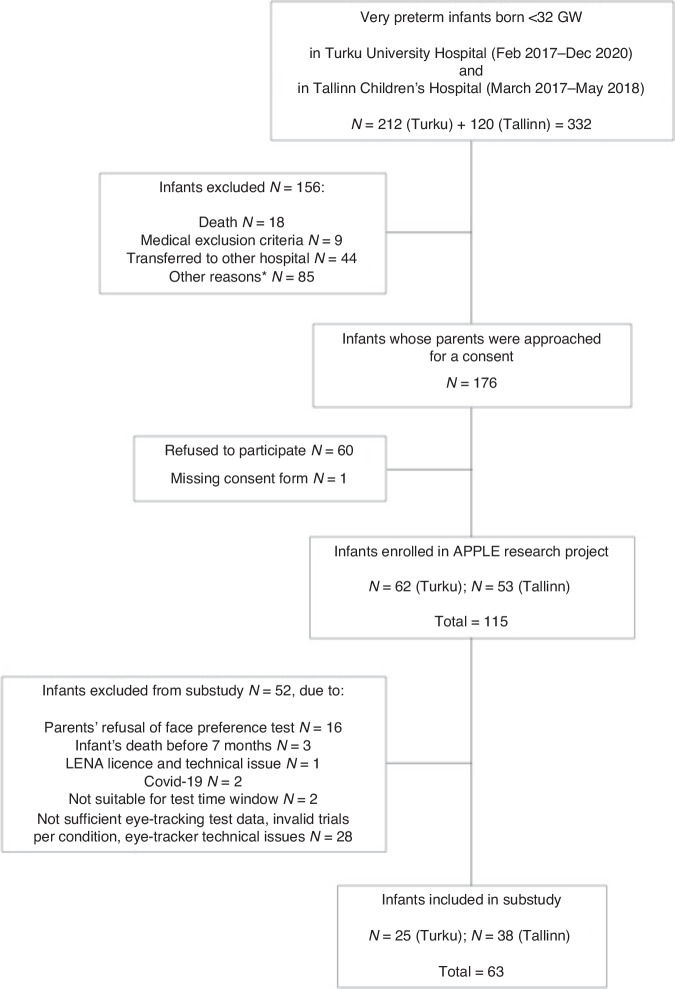
A flow chart. *Other reasons included license and technical issues, social reasons, native language, and multiple pregnancies of more than two fetuses.

The study population included preterm infants born before 32 weeks of gestation and their parents, speaking Finnish, Swedish, Estonian, or Russian. The exclusion criteria were a life-threatening disease, major congenital, chromosomal anomalies, and syndromes of clinical significance. The overall sample size in the APPLE Study was 115 preterm infants (62 infants from Turku and 53 infants from Tallinn). A total of 63 infants were included in the current study (Fig. 1. Flow chart).

### Protocol

The study protocol was approved by the Ethics Committees (the Ethics Committee, Hospital District of Southwest Finland, and the Research Ethics Committee of the University of Tartu, Estonia). The families got verbal and written information about the study and signed an informed consent form. Infant characteristics were collected from the hospital records after parental consent was acquired. The parents filled a questionnaire about family characteristics. Birth weight z-scores were calculated using Finnish growth references. Screening for retinopathy of prematurity was done for all patients in Tallinn and all patients born before 30 gestational weeks in Turku, according to the local guidelines.

Parents were asked to fill in the Closeness Diary^37^ for 14 days when their infant was at 32–34 weeks of postmenstrual age. The diary data included the duration of the parents’ presence in the unit, parent–infant skin-to-skin contact, and holding the dressed/clothed infant. A 16-hour recording of the auditory environment was performed at 32–34 weeks of postmenstrual age using Language Environment Analysis (LENA®, Boulder, Colorado). The LENA system classifies the auditory exposures into child vocalizations, female and male adult words, conversational turns, silence, TV/electronic sounds, and noise. The validity of the LENA system when used in Finnish and Estonian languages has been tested in the APPLE Study NICUs, which confirmed that it provides valid information on adult word count.^38^ During the recording, the microphone was placed near the infant’s head (within 10 cm from the head of the infant when the infant was in the cot or incubator and within 30 cm during skin-to-skin contact with his/her parent).

### Measurements

#### Exposure to the parents’ speech

The mother’s and father’s word counts were obtained from the LENA recordings using female and male word counts from the time intervals when each parent was present according to the diary data. We used the language environment data from the LENA recordings, using the hours between 7 a.m. and 10 p.m., as this was an active care time in the units. We calculated the exposure to the parents’ speech based on the word frequency (words/hour) when the parent was present during the recording day and the information on the parents’ presence during a two-week period. The parents’ presence was derived from the Closeness Diaries using only the hours between 7 a.m. and 10 p.m. Exposure to the parents’ speech = [total number of words when the parents were present during the analyzed recording/ total time the parents were present during the analyzed recording (hours)] x the parents’ presence (hours) for 14 days.

For parents who were not present during the recording day (two fathers in Turku, and one mother and ten fathers in Tallinn), the median mothers’ or fathers’ word frequency, separately from Turku and Tallinn, was used in the statistical analysis. When a parent was not present during the recording day for one of the twins, the word frequency registered during the other twin’s recording was used. In three cases, the mother was a single parent, and the exposure to the father’s speech was zero.

### Face preference

The infant’s face preference, using an eye-tracking based test, was conducted at the infant’s corrected age of seven months ( ± 7 days). In seven cases, the time window was exceeded (min –3 days and max +21 days) due to the infant’s acute illness or not finding a suitable time for the family.

During the testing, the infant sat on his/her parent’s lap at an approximately 60 cm distance from a 17-inch (1280×1024) computer screen. The test assessed the infant’s attention to faces and non-face patterns under conditions of distraction – that is, when a competing stimulus was added to the infant’s visual field.^23,24,26^ In this task, each trial began by the presentation of a fixation stimulus (a white circle or a ‘+’-sign) in the center of the monitor against a uniform black background. After the infant looked at the fixation stimulus, two stimuli were presented with a 1000-msec onset asynchrony. The first stimulus was a face or a non-face pattern, presented in the center of the screen (9.6° x 10.2°). The second stimulus was a black and white checkerboard pattern (a “distractor”), shown laterally ~12° left or right from the center for 2000 msec (3.5° x 12.7°). Each child saw a total of four 12-trial blocks in the test (i.e., 48 trials). In half of the trials, the first stimulus was a picture of a face displaying a neutral, happy, or fearful expression, posed by the infant’s parent (mother, father) or by an unfamiliar adult female model. In the other half of the trials, this stimulus was a luminance/color-matched pattern that was not identifiable as a face. The non-face patterns were created from each face stimuli by randomizing the phase spectra of the face image while retaining their amplitude and color spectra.^39^ The order of the face and non-face pattern trials was randomized for each child, with the constraint that each 12-trial block contained the same number of face and non-face trials. Parent faces were presented in blocks 2 and 3, and the faces of two unfamiliar female adults in blocks 1 and 4. The order of the blocks was the same for every child. The side of the lateral “distractor” was chosen randomly for each trial, but the total number of trials with the distractor on the left and right side of the screen was balanced within each block and in the face and non-face conditions.

The infants’ moment-by-moment gaze data were recorded throughout the test using a 30-Hz video camera that was mounted on a tripod behind the computer monitor (Canon Legria, HFR806) and an infrared eye-tracking camera mounted at the bottom of the screen (Eye Tribe; Copenhagen, Denmark or Tobii X2-60 camera; Tobii Technology, Stockholm, Sweden). Following previous studies,^26,40^ the data were analyzed trial by trial to determine whether and when gaze disengagement (i.e., shift) from the central stimulus to the lateral distractor occurred within an analysis period that started after the onset of the lateral stimulus (150–1000 msec). All analyses were based on the video camera recordings, as technically valid eye-tracking data were missing for many of the participants. Importantly, gaze disengagement can be reliably extracted from either video or eye-tracking recordings, and the results obtained by these two methods are highly consistent.^40^

Disengagement data were extracted for trials that met the following inclusion criteria adapted for video-based coding^41–43^: (i) the length of the fixation on the central stimulus was sufficient (i.e., ≥75% of the time), (ii) valid video data were available to document whether gaze disengagement occurred during the analysis period, (iii) the gaze disengagement (if any) was not premature (i.e., started 150 msec after the onset of the lateral stimulus, and (iv) the gaze shift was directed toward the lateral stimulus. All infants with >2 valid trials per stimulus condition were retained in the final statistical analyses (63 out of the 87 infants who had analyzable data; the median number of valid trials in face and non-face conditions varied from 8 to 10). Following previous video-based analyses,^41,43^ the disengagement values (0, 1) were averaged across trials to obtain estimates of the probability of no disengagement for each stimulus condition (familiar face, unfamiliar face; familiar non-face, unfamiliar non-face) for each infant. A measure based on the latency of the disengagement (i.e., dwell time) was also calculated, but not used as the primary measure, given that the temporal resolution of the video-based analyses was lower (30 Hz) than the temporal resolution in eye-tracking-based analyses of dwell time (60 Hz or higher). Results based on dwell time are given in Supplementary results.

A preference for faces is indicated by a higher probability of no disengagement in the face than the non-face condition. As the present paradigm was adopted from studies that have estimated disengagement probabilities separately for different facial expressions, we used neutral, happy, and fearful expressions as stimuli. However, recent research using this approach has shown that disengagement estimates are strongly correlated for neutral, happy, and fearful expressions and there is no evidence for unique individual variance for specific expression categories.^24,26^ For these reasons and to maximize the number of trials within each condition, we followed our a priori plan and averaged the data across different facial expressions.

### Data analysis

Descriptive statistics were used to describe the background characteristics. The differences between the study sites in word count and parents’ presence were tested using the Wilcoxon rank-sum test. As some of the outcome variables were not normally distributed and could not be normalized using common transformations, the pairwise differences were analyzed using the Wilcoxon test (effect sizes were estimated as z/sqrt(N)) and the correlations using Spearman rank correlations (r_s_). In the hypothesis-testing analyses, we calculated Spearman’s partial rank correlation coefficients (*r*_s_) between the variables describing the exposure to the parents’ speech and the mean proportion of no disengagement in the face condition, adjusted for the proportion of no disengagement in the non-face condition. To examine whether these correlations were affected by the covariates, we calculated partial r_s_ adjusted for gestational age, parental education, delivery type, sex, and birth weight. In an analysis testing the association between exposure to the parents’ speech and parent face preference, correlations between exposure to the parents’ speech and the proportion of no disengagement in the parent face condition were calculated, adjusted for the proportion of no disengagement in the unfamiliar face condition. The analyses and data visualizations were performed using the R and pResiduals, RVAideMemoire, dplyr, and ggplot2 packages.^44–48^

## Results

### Background of the study population

A total of 63 preterm infants and their parents (63 mothers and 60 fathers) were included in the analyses. The mean gestational age of the infants was 28^2/7^ weeks in Turku and 28^1/7^ weeks in Tallinn. The infant and family characteristics are described in Table 1.

**Table 1:** Infant and family characteristics.

	Turku *n* = 25	Tallinn *n* = 38
**GA at birth (weeks), mean (min;max)**	28^2/7^ (23^4/7^;31^6/7^)	28^1/7^ (24^5/7^;31^4/7^)
**Sex**		
Male	18 (72%)	21 (55%)
**Birth weight (g), mean**	1074	1118
**Delivery**		
Cesarean section	14 (56%)	22 (58%)
**Multiple**		
Single	18 (72%)	24 (63%)
Twin	7 (28%)	14 (37%)
**BPD at 36 PMA, of which**	12 (48%)	22 (58%)
Mild	1 (8%)	18 (82%)
Moderate	7 (58%)	4 (18%)
Severe	4 (34%)	0
**IVH**		
Grade III	0	1 (3%)
Grade IV	0	1 (3%)
**Cystic PVL**	0	2 (5%)
**Treated ROP**	0	2 (5%)
**Operated NEC**	0	0
**Positive blood culture sepsis**	1 (4%)	10 (26%)*
**Hearing status**		
Normal	24 (96%)	36 (94%)
Pathological	1 (4%)	1 (3%)
Unknown	0	1 (3%)
**Hearing aid needed at 1 year of corrected age**	0	0
**Maternal age (year), mean**	32.0	32.7
**Paternal age (year), mean**	34.0	34.3, unknown 2 (5%)
**Maternal education level, %**		
Basic education	2 (8%)	8 (21%)
General upper secondary school or vocational education and training	3 (12%)	11 (29%)
University of applied sciences	8 (32%)	3 (8%)
University	12 (48%)	16 (42%)
**Paternal education level, %**		
Basic education	4 (16%)	12 (32%)
General upper secondary school or vocational education and training	10 (40%)	10 (26%)
University of Applied Sciences	5 (20%)	2 (5%)
University	5 (20%)	12 (32%)
Unknown	1 (4%)	2 (5%)
**Maternal employment, %**		
Paid work	19 (76%)	33 (87%)
Unemployed	4 (16%)	4 (10%)
Student	2 (8%)	0
Unknown	0	1 (3%)
**Paternal employment, %**		
Paid work	25 (100%)	36 (95%)
Unknown		2 (5%)
**The mother’s native language, %**		
Estonian	0	28 (74%)
Russian	0	10 (26%)
Finnish	25 (100%)	0
**The father’s native language, %**		
Estonian	2 (8%)	29 (76%)
Russian	0	7 (19%)
Finnish	23 (92%)	0
Unknown	0	2 (5%)
**Sibling living at home, yes %**	12 (48%)	17 (45%)
**Previous child in the NICU, yes %**	3 (12%)	1 (3%)

Data are shown as absolute numbers (percentage).

*p value < 0.05.

*GA* gestational age, *BPD* bronchopulmonary dysplasia, *PMA* postmenstrual age, *IVH* intraventricular hemorrhage, *PVL* periventricular leukomalacia, *ROP* retinopathy of prematurity, *NEC* necrotizing enterocolitis, *NICU* neonatal intensive care unit.

The LENA recordings were performed at a mean age of 33^2/7^ weeks of postmenstrual age (SD 4 days) at both study sites. During the recording day, most infants were treated in a single-family room in Turku, and a room with two to four patients in Tallinn. None of the infants were in an incubator in Turku, whereas 45% were in an incubator in Tallinn. All infants needed a feeding tube to support enteral feeding. The environmental context during the recording day is presented in Table 2.

**Table 2:** Environmental context during the recording day.

	Turku (*n* = 25)	Tallinn (*n* = 38)
**Patients per room**		
1	16 (64%)	3 (8%)
2	9 (36%)	14 (37%)
3	0 (0%)	6 (16%)
4	0 (0%)	15 (39%)
**Temperature control**		
Incubator	0 (0%)	17 (45%)
Thermal support	6 (24%)	13 (34%)
None	19 (76%)	8 (21%)
**Breathing support**		
Invasive ventilation	4 (16%)	0 (0%)
CPAP/NIV-NAVA	5 (20%)	2 (5%)
High Flow Nasal Cannula	10 (40%)	16 (42%)
None	6 (24%)	20 (53%)

Data are shown as absolute numbers (percentage).

*CPAP* continuous positive airway pressure, *NIV-NAVA* noninvasive neutrally adjusted ventilatory assist.

### Exposure to the parents’ speech

During the recording day, the median word frequency (words per hour) was 1112 for the mothers and 329 for the fathers in Turku, and 232 and 14 words, respectively, in Tallinn. The word frequencies were significantly different between the study sites for the mothers (*p* < 0.001) and the fathers (*p* < 0.001).

The median of hours when the parent was present during the recording day between 7 a.m. and 10 p.m. was 8.3 hours for mothers (8.0 hours in Turku and 8.9 hours in Tallinn) and 1.3 hours for fathers (5.4 hours in Turku and 0.5 hours in Tallinn). The median of the parents’ presence during a period of two weeks, between 7 a.m. and 10 p.m., was 111 hours for the mothers and 58 hours for the fathers in Turku, and 177 and 13 hours, respectively, in Tallinn. The parents’ presence in the unit during the two-week period was significantly different between the study sites for the mothers (*p* < 0.001) and the fathers (*p* < 0.001).

**Fig. 2:**
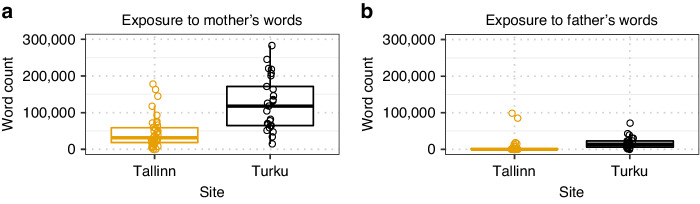
Exposure to the parents’ speech. Mothers (**a**), fathers (**b**), Tallinn (yellow), Turku (black).

When the word frequency was multiplied by the number of hours the parents were present for two weeks, the calculated exposure to the parents’ speech resulted in a median of 118,096 and 13,440 words per two weeks for the mothers and fathers, respectively, in Turku and a median of 31,146 and 151 words in Tallinn (Fig. 2). The exposures to the parents’ speech were significantly different between the study sites for the mothers (*p* < 0.001) and the fathers (*p* < 0.001).

### Exposure to the parents’ speech and face preference

**Fig. 3:**
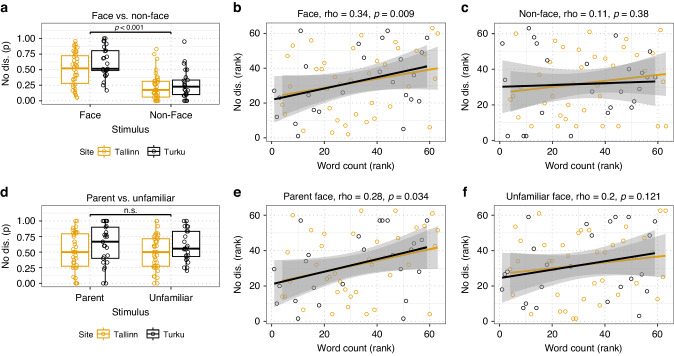
Exposure to the parents’ speech and face preference. The probability of no disengagement (No dis. (p)) was higher for faces than non-faces (**a**). Exposure to the parents’ speech was positively associated with the probability of no disengagement in the face condition (**b**) but not in the non-face conditions (**c**). The probabilities of no disengagement for parents and unfamiliar faces (**d**) were not statistically significant. Exposure to the parents’ speech was positively correlated with the probability of no disengagement for parent (**e**), but not for unfamiliar (**f**) adult faces. Ranked values are shown in **b**, **c**, **e**, and **f**.

The probability of no disengagement was higher in the face condition (*M* = 0.55, SD = 0.26) than in the non-face condition (*M* = 0.24, SD = 0.22), z = 6.7, *p* < 0.001, effect size = 0.84, demonstrating a preference for faces (Fig. 3a). The mean probability of no disengagement in the face condition was 0.62 (SD = 0.24) in Turku and 0.50 (SD = 0.27) in Tallinn, *p* = 0.10. The probability of no disengagement in the non-face condition was 0.26 (SD = 0.23) in Turku and 0.22 (SD = 0.21) in Tallinn, *p* = 0.52.

Given the large difference between the sites in word counts and a trend for a difference in disengagement probability for faces (*p* = 0.10), we adjusted the variables for the site difference for all subsequent analyses by transforming the variables into z-scores for each site separately. Total exposure to the parents’ speech (mother + father) was positively correlated with face preference, *r*_s_ = 0.34 [0.09 0.54], *p* = 0.009 (Fig. 3b). In separate analyses, the exposure to the mother’s speech correlated significantly with face preference, *r*_s_ = 0.32 [0.07 0.53], *p* = 0.01, whereas exposure to the father’s speech was not significantly associated with face preference, *r*_s_ = 0.20 [−0.06, 0.43], *p* = 0.14.

In adjusted analyses, the association between total exposure to the parents’ speech and infant face preference remained significant when controlling for the overall number of valid test trials or the age at test, *r*_s_ = 0.32–0.33, *p* < 0.05. Face preference was positively associated with gestational age, *r*_s_ = 0.27, [0.01, 0.50], *p* = 0.04, and negatively associated with the mother’s education, *r*_s_ = -0.26, [−0.47, −0.02], *p* = 0.03, but not with other covariates. The relation between exposure to the parents’ speech and infant face preference remained in an analysis adjusted for the covariates, *r*_s_ = 0.31 [0.04, 0.53], *p* = 0.025. This association was also observed when infant face preference was quantified by using disengagement latencies rather than disengagement probabilities (Supplementary results).

Because our data included a subset of dyads with family clustering (7 pairs of twins), we conducted a further sensitivity analysis to examine whether the main result replicated with subsets that included infants without a twin pair in the data as well as one individual infant from each twin pair. This analysis was repeated for all possible subsets, based on different possible ways of selecting unrelated individuals from the 7 pairs of twins (i.e., for 128 subsets). The correlation coefficient (*r*_s_) between total exposure to the parents’ speech and face preference ranged from 0.29 to 0.42 between the subsets, with a median value of 0.36.

### Exposure to the parents’ speech and parent face preference

The probability of no disengagement did not differ for parent (*M* = 0.55, SD = 0.31) versus unfamiliar (*M* = 0.54, SD = 0.27) adult faces (Fig. 3d). Thus, there was no preference for the parents’ faces over unfamiliar faces. Total exposure to the parents’ speech was correlated with parent face preference, *r*_s_ = 0.28 [0.02 0.49], *p* = 0.034 (Fig. 3e). This association was marginal when the infants without pictures of both parents (*n* = 5) were excluded from the analyses, *r*_*s*_ = 0.23 [−0.04 0.47], *p* = 0.096, or in analyses of subsets of infants without twin pairs (range 0.21–0.38, Med = 0.29). The correlation between total exposure to parents’ speech and parent face preference was similar when the preference was quantified by using a latency-based measure rather than a probability-based measure (Supplementary results).

## Discussion

The main finding of our study is that exposure to the parents’ speech during neonatal hospital care is associated with a stronger perceptual preference for faces at seven months of corrected age in preterm infants. This association suggests that exposure to the parents’ speech during neonatal hospital care can potentially be an important early marker for later social development.

We estimated exposure to the parents’ speech over a two-week period by using data from LENA recordings and diaries of the parents’ presence. This approach expanded the time window compared to earlier studies although it still provides only a sample of the continuous language exposure of the child. Caskey et al.^49^ reported a median of 567 adult words per hour at 32 weeks and 953 adult words per hour at 36 weeks of gestational age when a parent was visiting. There were more adult words and infant vocalizations in their open-bay NICU when a parent was present and a higher amount of parental talk was associated with better cognitive and language scores at seven and 18 months of corrected age.^16^ We found a comparable median number of the parents’ words per hour (494) when they were visiting their baby in the unit at 33 weeks of postmenstrual age. However, we found that the parental word count per hour (during the recording day) at the same age point was significantly higher (1441) in the NICU mostly consisting of single-family rooms (SFR) than in the NICU consisting of 2–4-infant rooms (246). A recent study by Aita et al found significantly lower sound levels in SFR,^50^ which protects infants from harmful noise exposure. Additionally, the positive effects of an SFR design on the parents’ presence^51,52^ and psychological wellbeing^52,53^ may explain why the parental word count was higher in the SFR unit. Moreover, in our study in the SFR unit, fewer infants were placed in incubators, potentially affecting parents’ communication with their infants. We therefore speculate that providing a calm environment with privacy may encourage families to talk more with their premature infants.

This is the first study to show an association between parental vocal contact and infant face preference. Consistently with previous work,^24–26^ our results showed that infants are less likely to disengage from faces than other patterns when “distracted” by a high-contrast lateral stimulus. This preference was positively correlated with gestational age. More mature preterm infants have a lower risk for atypical development and face preference seems to behave similarly. Previous studies have shown that compared to term infants, preterm infants (gestational age at birth between 23^2/7^–33^0/7^ weeks) looked less at the eyes vs. the mouth and less at the faces/people vs. non-social content.^18^ Another previous study showed no difference in disengagement from faces vs. patterns in moderate to late preterm, early-term, and full-term infants.^54^ Collectively, these results suggest that extreme and very preterm birth may be associated with reduced “preference” to attend to faces. The functional and developmental significance of these results, and the variations in face preference in general, are still poorly understood. The fact that this bias is a salient aspect of early development in infants suggests that it may have a central organizing role in early brain development, and possibly in the functional specialization of the brain for social cognition.^55^ This idea is supported by data indicating that the preference for faces in infants is positively correlated with prosocial behavior at two years of age^26^ and lower reported levels of callous-unemotional behaviors at up to four years of age.^26,56^

Our findings suggest that parental vocal contact may provide an early marker for later social engagement. This result seems to differ from the effects of the total word count of all adults on some other domains of early child development as the total adult word count during the recording day was negatively associated with language processing skills.^17^ Even though we estimated the parental speech exposure over a two-week period, it is important to emphasize that this estimate may be a proxy for the continuous language exposure of the child over time until 7 months of corrected age. However, a recent randomized controlled study found that parent-initiated language enrichment in the NICU improved language development of very preterm infants at 2 years of corrected age, thereby giving evidence for a causal effect.^34^ The other alternative is that rather than reflecting a causal effect established during NICU care, the correlation between parental speech exposure and infant face preference depends on the total amount of face-to-face contact and language exposure during the period of early child development and can also be mediated by other factor(s) that affect parental speech and infant development. Nevertheless, parental vocal contact may still provide a useful marker for early social engagement and those parents who do not talk much to/with their infant during NICU care, may benefit from tailored support for the early interaction.

Parental vocal contact with the infant may increase the salience of faces to infants as well as face-to-face interaction, both of which are crucial for early learning for infants. Our results showing that parental vocal contact was associated with overall face preference and parent face preference suggest that exposure to parental vocal contact increases the infants’ social engagement in general and with parents specifically. We were not able to compare the relative strengths of the associations with general vs. parent face preference, however, as the estimates for the two were based on different number of trials. Given the limited sample, we also had to pool the data from the two sites and were not able to perform separate analyses by site to assess whether the reported associations varied by site. Further research is needed to address these questions. Further research is also needed to examine whether the unexpected negative correlation between maternal education and child face preference replicates in other samples.

Exposure to the fathers’ speech was not consistently associated with face preference whereas exposure to the mothers’ speech was. The explanation might be that the fathers were less often present, and therefore the infant heard fewer paternal words in the NICU. In our study, infants were exposed significantly more to the fathers’ speech in the unit with SFR than in the unit with mainly open-bay rooms. Providing a calm environment and privacy could encourage fathers to talk more with their infants. We highlight the need to promote paternal participation in NICUs, and further studies about paternal vocal contact are needed.

The key strengths of this study are the inclusion of direct, observational measures of adult and child behavior, as well as the inclusion of language environment data from two units with different care cultures and architecture. Additionally, including the data from both parents from a two-week period allowed us to broaden the time window for vocal contact. This study also has limitations. The identification of the speakers was not possible from the audio recordings, so it is likely that the word count may, in some cases, also include parent-staff discussions near the infant. However, we do not expect neighboring parents’ speech to be included in LENA word counts which only includes near and clear speakers. Of note, this study was intended to measure speech near the infant, not infant-directed speech. The LENA recordings started mainly at the same time in the morning, but some of the recordings started later due to technical reasons, which could have influenced the word count. We calculated the parental presence and word counts considering different recording starting points to get the most comparable data. Another limitation of the study is that some fathers and one mother were not present during the recording day and therefore we chose to use the median word frequency to give the best estimate of the word count for the time they were present during the diary days. The estimation was done to provide a wider time window for the exposure to parents’ speech compared to only one recording day. For example, if the father was not present during the recording day, his words would have been 0 if we had not used the median words of the sample for the hours he was present. Finally, while the auditory environment recording was feasible and succeeded well, the eye-tracking test was challenging and was not carried out successfully for 33% of the participants due to technical difficulties. Therefore, numerous patients were recruited to have a sufficient study sample. For future studies, it would be important to investigate the long-term impact of parental vocal contact on a child’s neurodevelopment in larger populations.

## Conclusion

The results demonstrate that higher exposure to the parents’ speech is associated with better social-cognitive development as measured by an attentional preference for faces over non-face patterns. This adds to the literature suggesting that the parents’ vocal contact with their preterm infant is a correlate and potentially a supportive factor of early childhood development.

## Supplementary information

Supplementary Information

## Data Availability

The datasets generated and analyzed during the current study are not publicly available due to confidentiality reasons as parents’ words have been recorded (LENA), but are available from the corresponding author on reasonable request.
