# Supplementary material for: Exposure to the parents’ speech is positively associated with preterm infant’s face preference
Source: Pediatr Res. 2024 May 23;96(7):1803–11. doi: 10.1038/s41390-024-03239-8 (PMC11772228; doi:10.1038/s41390-024-03239-8)
Supplement: Supplementary file 1 — Supplementary Information [file 41390_2024_3239_MOESM1_ESM.pdf]

Supplementary results

In supplementary analyses, infant face preference was quantified by using disengagement latencies rather than disengagement probabilities. Disengagement latencies were converted into normalized dwell time indices by using the formula described in Leppänen et al. (2015). The shortest acceptable disengagement latency (150 ms) resulted in a dwell time index of 0, and the longest possible latency (or a lack of disengagement, which is equal to the last measured time point at 1000 ms) in a dwell time index of 1.

Results are shown in Supplementary Figure 1. Dwell times were longer for faces (M = 0.70, SD = 0.20) than non-face patterns (M = 0.43, SD = 0.19), z = 6.8, p < 0.001, effect size = 0.85. Total exposure to the parents' speech was positively correlated with dwell time on faces, adjusted for dwell time on non-face patterns, r_s = 0.27 [0.03 0.49], p = 0.030. Dwell times did not differ for parent (M = 0.69, SD = 0.23) and unfamiliar (M = 0.70, SD = 0.20) faces, p = .580. Total exposure to the parents' speech was positively correlated with dwell time for parent faces, adjusted for dwell time for unfamiliar faces, r_s = 0.27 [0.02 0.50], p = 0.038.

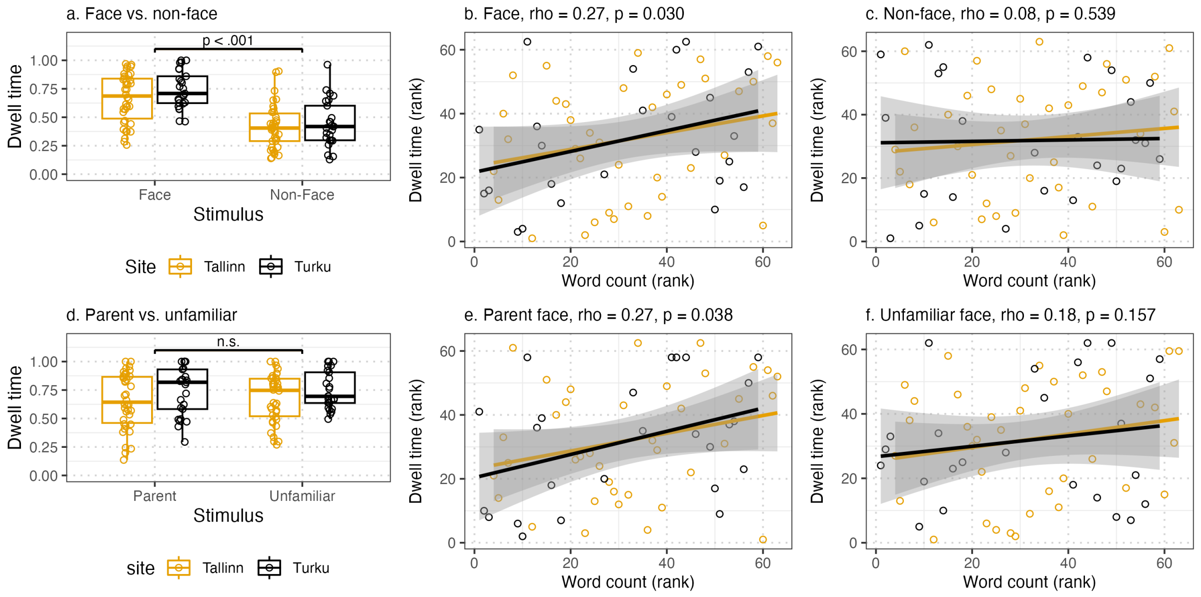

Supplementary Figure 1. Exposure to the parents' speech and dwell times for faces and non-faces. Dwell times were longer for faces than non-faces (a). Exposure to the parents' speech was positively associated with dwell time for faces (b) but not with dwell time for non-faces (c). Dwell times did not differ for parent and unfamiliar faces (d). Exposure to the parents' speech was positively correlated with dwell time for parent (e), but not for unfamiliar (f) adult faces. Ranked values are shown in b, c, e, and f.

References

Leppänen, J. M., Forssman, L., Kaatiala, J., Yrttiaho, S. & Wass, S. Widely applicable MATLAB routines for automated analysis of saccadic reaction times. *Behav. Res. Methods* 47, 538–548 (2015).
